# The Double Standard of Ownership

**DOI:** 10.1162/opmi_a_00190

**Published:** 2025-02-16

**Authors:** Zofia Washington, Ori Friedman

**Affiliations:** Department of Psychology, University of Waterloo, Waterloo, Canada

**Keywords:** praise-blame asymmetry, ownership, moral agency, vicarious responsibility

## Abstract

Owners are often blamed when their property causes harm but might not receive corresponding praise when their property does good. This suggests a double standard of ownership, wherein owning property poses risks for moral blame that are not balanced with equal opportunities for credit. We investigated this possibility in three preregistered experiments on 746 US residents. Participants read vignettes where agentic property (e.g., animals, robots) produced bad or good outcomes, and judged whether owners and the property were morally responsible. With bad outcomes, participants assigned owners more blame than property (Experiments 1 and 2) or similar blame (Experiment 3). But with good outcomes, participants consistently assigned owners much less praise relative to their property. The first two experiments also examined if the double standard arises in two other relationships between authorities and subordinates; participants showed the double standard when assessing moral responsibility for parents and children, but not for employers and employees. Together, these findings point to a novel asymmetry in how owners are assigned responsibility.

## INTRODUCTION

In all 50 states, dog owners are legally responsible for injuries caused by their dogs (Bieber, 2023). According to Dog Bite Laws, when a dog causes harm the owner holds the weight of liability, not the animal. On the other hand, when a dog acts heroically, there are medals, awards, and memorials that exist solely for honoring and celebrating it, such as the Dicken Medal for Gallantry and the Animals in War & Peace Medal of Bravery. One recent recipient is Kuno, a dog that tackled an enemy gunman in Afghanistan, saving the lives of British soldiers (BBC News, 2020). Media coverage of the event makes barely any mention of Kuno’s handler, giving praise solely to the animal.

These examples suggest a double standard of ownership where owning property poses risks for moral blame that are not balanced with equal opportunities for credit. In this double standard, owners receive at least as much blame as their property when it causes harm, but they do not receive equal praise when property produces good outcomes. This double standard—if people show it—would reveal a dark side to owning agentic property that can act on its own, like a dog or a robot. Owning things is often assumed to be desirable. For example, many theorists suggest that ownership rules arise because of the need to regulate access to desirable resources (e.g., Boyer, 2023; Ellickson, 1989; Stake, 2004). Liability for harm caused by their property is one potential drawback to owning agentic property. This drawback would be greater if people show the double standard of ownership, and their tendency to hold owners liable for harm caused by property is not matched by corresponding credit to owners for the good outcomes their property produces.

The double standard is also relevant for our understanding of moral responsibility. Much previous work has revealed differences between how people assign blame and praise. One example is the side-effect effect (Knobe, 2003). People see a company owner as blameworthy if he indirectly harms the environment, but not as praiseworthy if he indirectly benefits it. Beyond this, people give more weight to intentionality and other mental states when evaluating morally bad than good acts (Guglielmo & Malle, 2019; Pizarro et al., 2003); they participate in more causal reasoning for bad outcomes than good outcomes (Bohner et al., 1988; Chu & Shaw, 2005); and when considering group processes, they narrowly blame individual group members for failure while broadly praising the overall groups for success (Schein et al., 2020; for a review see Anderson et al., 2020). The double standard of ownership, then, may be another praise-blame asymmetry.

Why might people show this double standard of ownership? When people identify harmful outcomes, they search for the agent who directly caused the harm and who can be blamed for it (Cushman, 2008; Gray et al., 2012; Malle et al., 2014). However, sometimes people also blame other agents who did not directly cause the harm. Such vicarious blame is often assigned to agents in authority positions (e.g., Hamilton, 1986; Hamilton & Sanders, 1981, 1995; Shultz et al., 1987). For example, in one study, participants read a story where one construction worker caused an accident after his co-worker neglected to warn him about potential danger. Participants viewed the co-worker more negatively when he was the other worker’s boss rather than the other worker’s peer or employee (Haidt & Baron, 1996). Thus, people often trace moral blame back to agents who did not directly produce the harm, but who had an obligation to prevent it (Malle et al., 2014).

Owners are common targets for such vicarious blame. They are often held responsible for harm caused by pets, livestock, and other possessions (e.g., Chiu & Hong, 1992; Nadler & McDonnell, 2011). For example, if a dog escapes from its owner’s property and mauls a child, people hold the dog owner responsible. Assigning owners responsibility for harm caused by their property was codified in ancient law from both the Near East and China (Sznycer & Patrick, 2020), and is also seen in young children (Bowman-Smith et al., 2018). However, owners might *not* receive corresponding vicarious praise when their property does good. While seeing harm prompts people to search for a moral agent to blame (Cushman, 2008; Gray et al., 2012; Malle et al., 2014), seeing good might not prompt search for such an agent to credit. Also, while people often withhold blame from agents that lack certain mental abilities (Gray et al., 2007), they may nonetheless be willing to praise these agents; as discussed above, people give less weight to intentionality and other mental states when evaluating morally good than bad acts (Anderson et al., 2020). So, when agentic property (e.g., a dog or a robot) produces a good outcome, people may praise it and pay little attention to its owner.

We investigated whether people show a double standard of ownership in three experiments. In each experiment, participants read vignettes where agentic property produces bad or good outcomes. Participants then rated whether the owner and the agentic property each deserved blame or praise (depending on the outcome valence). We looked at two examples of agentic property—pets and robots. People have owned animals for millennia (e.g., Ellickson, 2013) whereas owning robots is newer and increasingly relevant in an age of self-driving cars and AI software. Some work suggests that people see robots as having less agency than humans, but more agency than dogs (Gray et al., 2007; Gray & Wegner, 2012). This may result in a stronger double standard effect for pets than owned robots.

In the first two experiments, we also looked at moral responsibility in two other authority-based relations—parents’ responsibility for their children, and employers’ responsibility for employees. These relations may offer informative comparisons with ownership. Much as owners are liable for harm produced by property, parents are seen as somewhat blameworthy or responsible when their children commit crimes, especially when the children are younger rather than older (e.g., Aizpurua et al., 2020; Brank et al., 2011). Also, while people do not see children as owned (Starmans & Friedman, 2016), they might see them as resembling owned property in some ways, since they might see children as lacking full autonomy and moral agency (Gray & Wegner, 2009).

Turning to workplace relations, employers and managers are often blamed when employees under their authority cause harm (e.g., Haidt & Baron, 1996) and receive the bulk of the blame when their companies produce disappointing outcomes (e.g., Schein et al., 2020, Study 3; also see Palmeira et al., 2023). In fact, one experiment on workplaces is suggestive of something like the double standard of ownership. Participants predominantly blamed high-level employees for corporate failure, but predominantly praised low-level employees for success (Gibson & Schroeder, 2003). But in contrast with children (and agentic property), employees are likely to be viewed as true moral agents—after all, they are regular adults.

## GENERAL METHOD & TRANSPARENCY AND OPENNESS

Preregistrations, materials, data, and code for all experiments are available on OSF at https://osf.io/64zpu/. We disclose all measures, manipulations, and exclusions.

Participants were residents of the United States and tested using Qualtrics and CloudResearch. They had a HIT approval rate of 95–100% over at least 100 prior HITs and we used the “block low quality participants” option. After Experiment 1, participation was limited to individuals who had not completed a prior experiment in the series. Each experiment concluded with multiple-choice attention checks (see the materials at OSF), which were asked to ensure participants had read the vignettes. As preregistered, we excluded participants who failed one or more of these questions. After these questions, participants were also asked about their age and gender.

In each experiment, we sought to recruit approximately 100 participants per between-subjects condition; we based this goal on previous studies using similar designs. Our final samples in Experiments 1 and 2 fell short of this goal because more participants failed comprehension checks than anticipated.

The main analyses were run in R. In each experiment, we used the “geepack” package (Højsgaard et al., 2006) to run a generalized estimating equations (GEE) model for linear data and “emmeans” (Lenth, 2023) to run pairwise tests comparing responses across conditions. We also report sensitivity power analyses conducted using the PANGEA webtool (Westfall, 2015) available at https://jakewestfall.shinyapps.io/pangea/. We ran each analysis twice, testing the power to detect both a medium effect (*d* = 0.45) and a small effect (*d* = 0.20). The power analyses report the power to detect the highest-order effect in each design; the Supplemental Materials show the details of these analyses.

## EXPERIMENT 1

### Methods

#### Participants.

We tested 165 participants (*M*_age_ = 39 years; 60 women, 104 men, 1 preferring not to answer); 57 more were excluded. With this sample, we could detect a 2-way interaction (i.e., the highest-order effect in our design) with >99% power for a medium effect and 66% power for a small effect, both at *α* = 0.05.

#### Procedure.

**Figure 1.**
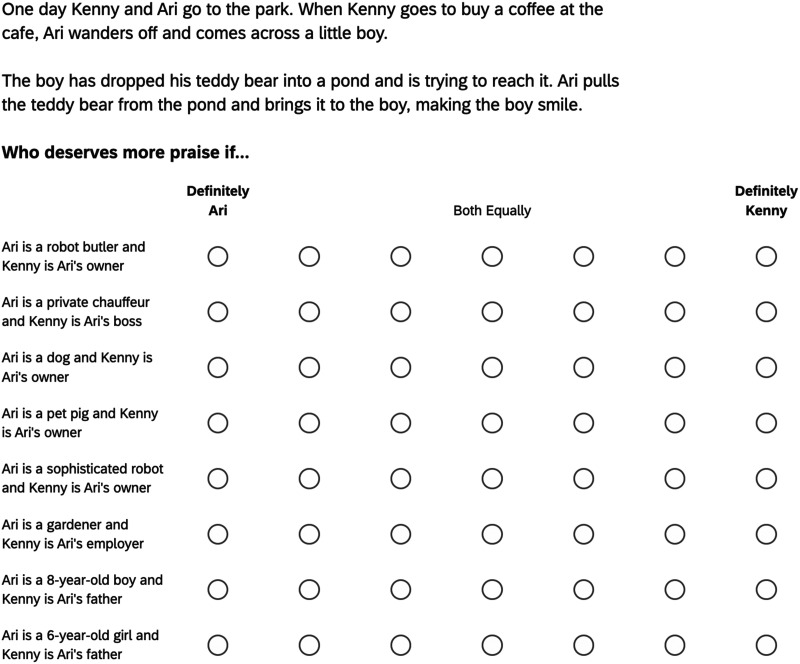
Sample trial from Experiment 1. *Note*. The order in which relationship trials appear in the matrix table was randomized across participants.

Participants read a story about two characters—an authority Kenny, and his subordinate Ari. Both characters were at a park, but Ari wandered off and interacted with a little boy who had dropped his teddy bear in a pond. After reading the story, participants indicated which of the characters deserved more blame or praise in 8 circumstances listed on a single screen (order randomized); see Figure 1.

The experiment used a 2 × 4 design. One factor was valence (between-subjects). In the good valence condition, the subordinate returned the teddy bear to the boy and participants rated which character deserved more praise; in the bad valence condition, the subordinate ran away with the teddy bear and participants rated which character deserved more blame. Responses were entered on a 7-point Likert scale with the anchors “Definitely Ari (1), “Both Equally” (4) and “Definitely Kenny” (7).

The other factor was the subordinate’s relationship to the authority (within-subjects). The subordinate was either the authority’s pet, robot, child, or employee. Each participant gave ratings for two trials per relationship. For example, the two trials for the pet relationship were Ari as a dog or as a pet pig.

### Results

**Figure 2.**
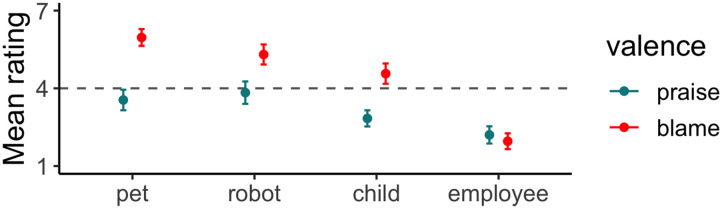
Mean ratings of praise and blame towards the authority over the subordinate for subordinate actions in Experiment 1; error bars depict 95% confidence intervals.

Figure 2 shows participants’ mean ratings. A 2 (valence: good, bad) by 4 (relation to authority: pet, robot, child, employee) GEE model revealed main effects of valence, *χ*^2^(1) = 53.20, *p* < .001, and relationship, *χ*^2^(3) = 333.12, *p* < .001, with a significant interaction between these factors, *χ*^2^(3) = 90.34, *p* < .001. Follow-up comparisons with *p*-values adjusted for 4 tests found that, when the subordinate was a pet, robot, and child, participants assigned relatively more responsibility to authorities than subordinates when assigning blame for negative outcomes than when assigning praise for positive outcomes: pet, *M*_difference_ = 2.41, *SE* = 0.26, Z_ratio_ = 9.24, *p* < .001; robot, *M*_difference_ = 1.47, *SE* = 0.30, Z_ratio_ = 4.98, *p* < .001; and child, *M*_difference_ = 1.73, *SE* = 0.26, Z_ratio_ = 6.75, *p* < .001. However, for the employee relationship, there was no difference between praise and blame, *M*_difference_ = −0.25, *SE* = 0.23, Z_ratio_ = −1.06, *p* > .999 and overall participants mostly assigned both to the employee.

### Discussion

Together, these findings suggest a double standard of ownership, where owning property (and other kinds of authority-subordinate relationships) poses risks for blame not matched by opportunities for credit. Authorities received most of the blame when their subordinates caused harm, but this was *not* mirrored with praise when subordinates were helpful. The only exception to this pattern was in the relationship between an employer and an employee. Findings here may have differed because, unlike the other subordinates, the employee was a fully autonomous agent (i.e., an adult).

In the next experiment, we examined separate ratings for subordinates and authorities. The joint ratings of Experiment 1 assumed that participants wanted to blame or praise someone. However, in some scenarios participants may have felt that neither the authority nor the subordinate deserved much blame or praise at all. For example, with the joint ratings scale, a mid-point answer could mean low praise and blame for both authority and subordinate, or high praise and blame for each. Separate authority and subordinate ratings in Experiment 2 allowed us to better understand the pattern of results. We also used a new scenario. The authority in the current experiment might have been seen as negligent because he left the subordinate unattended in a public space. In Experiment 2, we attempted to reduce negligence by having the story take place in the authority’s gated backyard.

## EXPERIMENT 2

### Methods

#### Participants.

We tested 179 participants (*M*_age_ = 41 years; 75 women, 103 men, 1 preferring not to answer); 40 more were excluded. With this sample, we could detect a 3-way interaction (i.e., the highest-order effect) with >99% power for a medium effect and 54% power for a small effect, both at *α* = 0.05.

#### Procedure.

**Figure 3.**
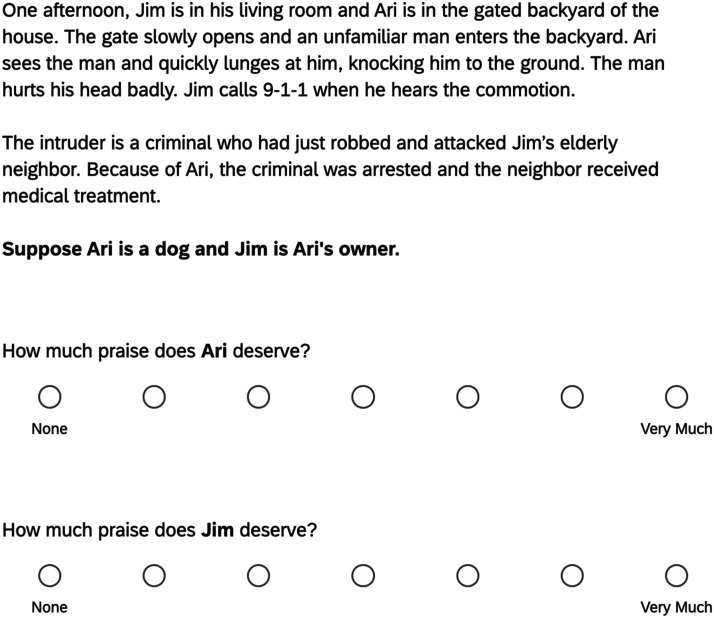
Sample trial from Experiment 2. *Note*. The order in which the two questions appeared was randomized across participants.

Participants read a story about two new characters, Ari and Jim. In the story, Ari attacked a man who entered Jim’s backyard; see Figure 3. Participants then morally evaluated both characters in four circumstances.

The experiment used a 2 × 4 × 2 design. One factor was again valence (between-subjects). In the good condition, the subordinate attacked a criminal and participants rated how much praise was deserved; in the bad condition, the intruder was an elderly neighbor with dementia and participants instead rated how much blame was deserved. This manipulation of valence was somewhat different than in the first experiment—while the subordinate in the first experiment performed different actions across the good and bad valences (i.e., returning a teddy bear or stealing it), in this experiment the action was the same (attacking the intruder). This change was made to make the valence conditions more symmetrical.

Another factor was, again, the subordinate relationship to the authority (within-subjects). As before, there were four relationships: pet, robot, child, and employee. However, each was examined on a separate screen (order randomized across participants). While in Experiment 1, the child was six or eight years old, in this experiment the child was eleven, because the child had to be plausibly able to knock down an adult intruder. Also, while the employee’s job in Experiment 1 was unrelated to the subordinate’s action, in this experiment the job was directly related—that is, the employee was a security guard and dealing with intruders is part of that job. This change was made to see if employers are held accountable when employees perform wrongs related to their duties.

The final factor was judgment target (within-subjects). For each subordinate to authority relationship, participants answered separate questions about both the subordinate and the authority. The question order was counterbalanced across participants and they responded on 7-point Likert scales with the anchors “None” (1) and “Very Much” (7).

### Results

**Figure 4.**
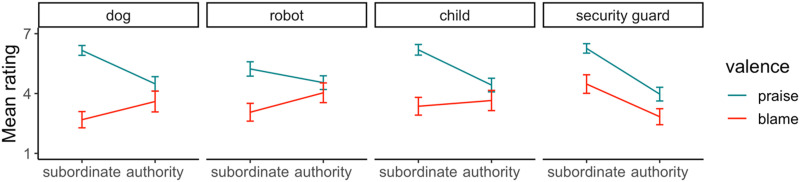
Mean ratings of praise and blame towards the authority and subordinate in Experiment 2; error bars depict 95% confidence intervals.

Figure 4 shows participants’ mean ratings. A 2 (valence: praise, blame) by 4 (relation to authority: pet, robot, child, employee) by 2 (judgment target: subordinate, authority) GEE model revealed a main effect of valence, *χ*^2^(1) = 74.49, *p* < .001, and a main effect of judgment, *χ*^2^(1) = 36.05, *p* < .001; the main effect of relationship was not significant, *χ*^2^(3) = 7.15, *p* = .067. The 2-way interactions were all significant: valence and relationship, *χ*^2^(3) = 22.87, *p* < .001, relationship and judgment, *χ*^2^(3) = 115.13, *p* < .001, and valence and judgment, *χ*^2^(1) = 50.42, *p* < .001. There was also a significant 3-way interaction, *χ*^2^(3) = 28.37, *p* < .001.

Follow-up comparisons with *p*-values adjusted for 8 tests revealed the double standard of ownership in three relationships. Participants assigned more blame to the owner than the pet (*M*_difference_ = 0.91, *SE* = 0.26, Z_ratio_ = 3.46, *p* = .004) and robot (*M*_difference_ = 0.98, *SE* = 0.33, Z_ratio_ = 2.99, *p* = .022), and comparable blame to the parent and its child (*M*_difference_ = 0.29, *SE* = 0.31, Z_ratio_ = 0.92, *p* > .999), but without assigning the authority more or comparable praise. Instead, the authority received *less* praise: pet, *M*_difference_ = 1.68, *SE* = 0.19, Z_ratio_ = 8.88, *p* < .001; robot, *M*_difference_ = 0.69, *SE* = 0.22, Z_ratio_ = 3.07, *p* = .017; child, *M*_difference_ = 1.77, *SE* = 0.20, Z_ratio_ = 9.08, *p* < .001. The employee relationship followed a different pattern as the employee received more blame (*M*_difference_ = 1.64, *SE* = 0.20, Z_ratio_ = 7.25, *p* < .001) and praise (*M*_difference_ = 2.29, *SE* = 0.20, Z_ratio_ = 11.55, *p* < .001) than the employer.

We also found that subordinates were praised much more than they were blamed: pet, *M*_difference_ = 3.47, *SE* = 0.24, Z_ratio_ = 14.28, *p* < .001; robot, *M*_difference_ = 2.17, *SE* = 0.29, Z_ratio_ = 7.41, *p* < .001; child, *M*_difference_ = 2.83, *SE* = 0.26, Z_ratio_ = 10.68, *p* < .001; employee, *M*_difference_ = 1.79, *SE* = 0.27, Z_ratio_ = 6.72, *p* < .001. However, the difference between praise and blame assigned for authorities was non-significant or at least smaller: pet owner, *M*_difference_ = 0.88, *SE* = 0.32, Z_ratio_ = 2.74, *p* = .050; robot owner, *M*_difference_ = 0.51, *SE* = 0.31, Z_ratio_ = 1.66, *p* = .784; parent, *M*_difference_ = 0.77, *SE* = 0.31, Z_ratio_ = 2.49, *p* = .103; employer, *M*_difference_ = 1.13, *SE* = 0.27, Z_ratio_ = 4.20, *p* < .001.

### Discussion

These findings are generally consistent with the results from our first experiment. Together, the findings suggest that, whereas authorities—especially owners—are often blamed when their subordinates caused harm, these same authorities do not receive correspondingly higher praise when subordinates bring about good outcomes. The findings also suggest that while people will assign authorities comparable levels of praise and blame, they are more willing to praise than blame subordinates.

One concern, though, is that praise for the subordinates might have been inflated. For example, participants might have lavished high praise on subordinates because they were surprised by dogs, robots, and children taking heroic actions (or at least, participants might have found this more surprising than the subordinates causing harm). On this account, authorities might have received comparatively less praise for the good acts of their subordinates because the surprisingness did not extend to them. In the next experiment, we were able to assess this possibility by adding scenarios where the authority was the one committing a moral action. Because the previous experiments both found similar patterns across three of the relationships (pet, robot, child), we only examined judgments about pet ownership in this experiment.

## EXPERIMENT 3

### Methods

#### Participants.

We tested 402 participants (*M*_age_ = 41 years; 191 women, 205 men, 6 preferring to self-identify to not to answer); 27 more were excluded. With this sample size, we could detect a 3-way interaction (i.e., the highest-order effect) with >99% power for a medium effect and 60% and 58% power for a small effect in our “kite” and “backyard” stories respectively (described below), both at *α* = 0.05.

#### Procedure.

**Figure 5.**
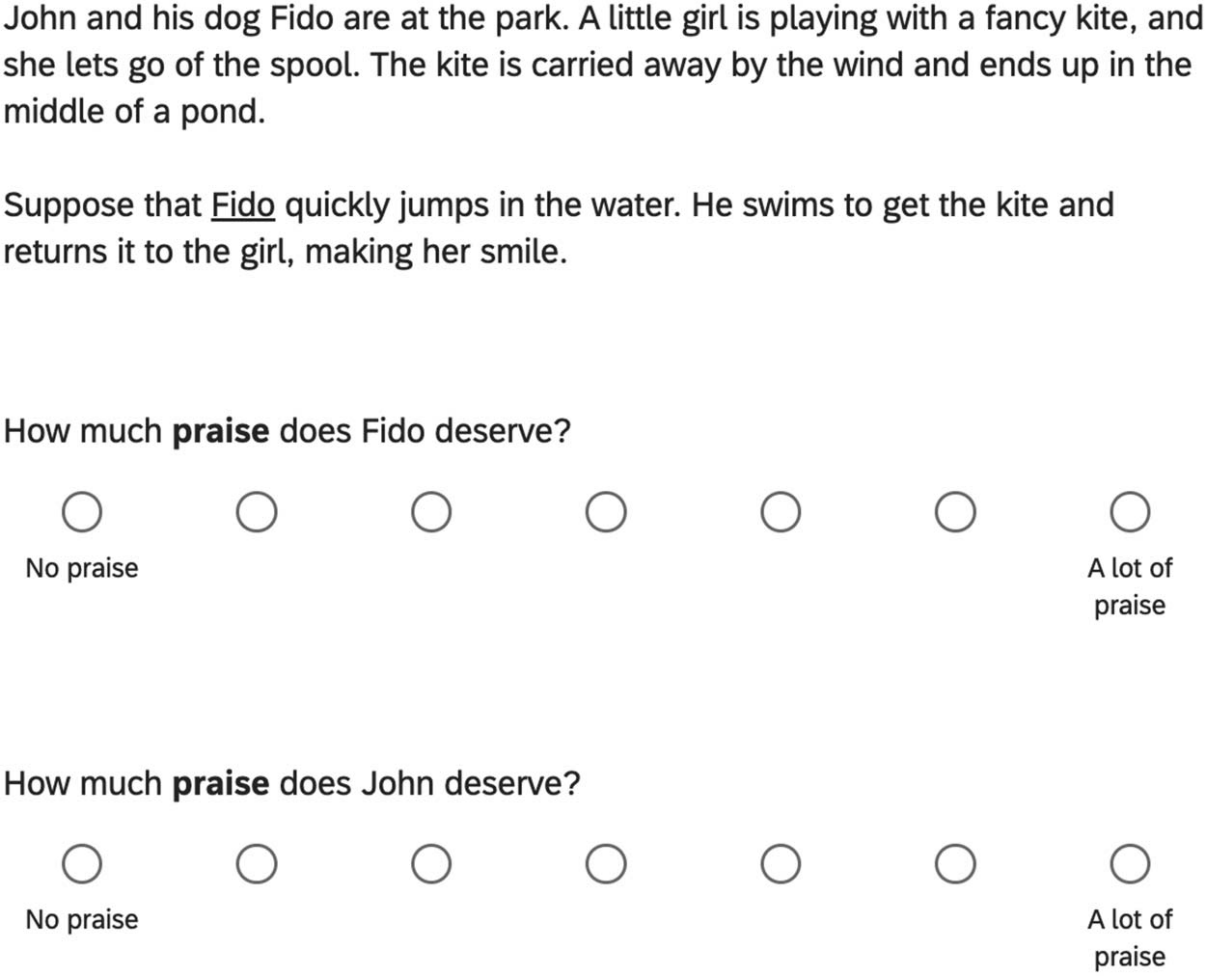
Kite story sample trial from Experiment 3. *Note*. Questions order was randomized across participants.

Participants read a story about two new characters, Fido the dog and Fido’s owner John. They first read the story preamble and were then informed that they would read and answer questions about two different endings. Participants were randomly assigned to either read a story where Fido and John saw a little girl’s kite get stuck in a pond (see Figure 5), or one where a man unexpectedly entered John’s backyard.

Besides this manipulation of story setting, the experiment used a 2 × 2 × 2 design. One factor was whether Fido or John was the principal actor in the story (between-subjects). Another factor was valence (within-subjects). Each participant read and provided ratings for two versions of a story—a good valence version where the principal actor was helpful and participants rated praise, and a bad valence version where the principal actor caused harm and participants rated blame (order randomized across participants). In the good valence versions, the actor returned the kite to the little girl (kite story) or attacked a criminal (backyard story) in the bad valence versions, the actor ran away with the kite (kite story) or attacked a senior with dementia (backyard).

The final factor was judgment (within-subjects). Participants answered two questions about each story ending, one about the dog and the other about the owner. The question order was counterbalanced across participants and participants responded to each of these questions on a 7-point Likert scale with anchors “No [praise/blame]” (1) and “A lot of [praise/blame]” (7).

### Results

**Figure 6.**
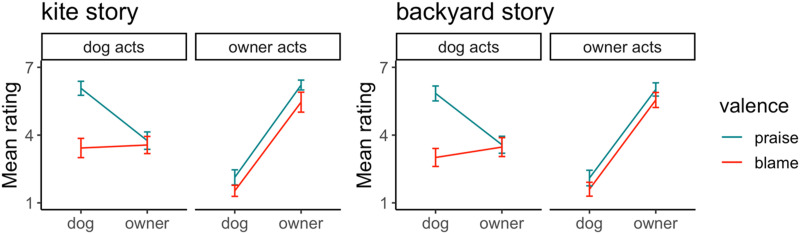
Mean rating scores of praise and blame towards a dog and its owner when either the dog or the owner commits an act; error bars depict 95% confidence intervals.

Figure 6 shows participants’ mean ratings. Two separate 2 (valence: praise, blame) by 2 (actor: dogs acts, owner acts) by 2 (judgment target: dog, owner) GEE models revealed that all main effects were significant, except for actor effect in the backyard story, and that all interactions were also significant, including the 3-way interactions between valence, actor, and judgment, both *p*s < .001; Table 1 lists all effects. To understand the 3-way interactions, we conducted separate valence × judgment analyses for each actor in both stories.

**Table 1.** Summary of 2 × 2 × 2 GEE Models in Experiment 3

Model terms	Kite story	Backyard story
Valence	*χ*^2^(1) = 84.26, *p* < .001	*χ*^2^(1) = 62.69, *p* < .001
Actor	*χ*^2^(1) = 6.32, *p* = .012	*χ*^2^(1) = 1.03, *p* = .310
Judgment	*χ*^2^(1) = 107.92, *p* < .001	*χ*^2^(1) = 128.05, *p* < .001
Valence * Actor	*χ*^2^(1) = 10.526, *p* = .001	*χ*^2^(1) = 16.13, *p* < .001
Valence * Judgment	*χ*^2^(1) = 34.02, *p* < .001	*χ*^2^(1) = 56.89, *p* < .001
Actor * Judgment	*χ*^2^(1) = 330.38, *p* < .001	*χ*^2^(1) = 327.10, *p* < .001
Valence * Actor * Judgment	*χ*^2^(1) = 44.21, *p* < .001	*χ*^2^(1) = 327.10, *p* < .001

#### Kite Story.

When the dog was the actor, there was a main effect of valence, *χ*^2^(1) = 57. 62, *p* < .001, and judgment, *χ*^2^ (1) = 30.89, *p* < .001, and a significant interaction between these factors, *χ*^2^(1) = 74.40, *p* < .001. Follow-up comparisons with *p*-values adjusted for 4 tests found it resulted because the owner received as much blame as the dog (*M*_difference_ = 0.13, *SE* = 0.27, Z_ratio_ = 0.50, *p* > .999), but much less praise (*M*_difference_ = 2.32, *SE* = 0.22, Z_ratio_ = 10.73, *p* < .001).

However, when the owner was the actor, there were significant main effects of valence, *χ*^2^(1) = 26.66, *p* < .001, and judgment, *χ*^2^(1) = 400.68, *p* < .001, but the interaction was not significant, *χ*^2^(1) = 0.35, *p* = .557. Participants gave higher ratings for praise than blame, and these ratings were much higher for the owner (i.e., who acted) than for the dog.

The findings also suggest that while participants were willing to lavish praise on the dog, they never assigned it comparable blame. For example, when the dog was the principal actor, the dog received much less blame than praise (*M*_difference_ = 2.64, *SE* = 0.25, Z_ratio_ = 10.71, *p* < .001). On the other hand, the owner received similar levels of vicarious blame and praise for their dog’s actions (*M*_difference_ = 0.19, *SE* = 0.22, Z_ratio_ = 0.87, *p* > .999).

#### Backyard Story.

When the dog was the actor, there was a main effect of valence, *χ*^2^(1) = 48.56, *p* < .001, and judgment, *χ*^2^(1) = 24.06, *p* < .001, and a significant interaction between these factors, *χ*^2^(1) = 100.51, *p* < .001. Follow-up comparisons with *p*-values adjusted for 4 tests found it resulted because the owner received the same blame as the dog (*M*_difference_ = 0.46, *SE* = 0.24, Z_ratio_ = 1.89, *p* = .237), but much less praise (*M*_difference_ = 2.27, *SE* = 0.22, Z_ratio_ = 10.55, *p* < .001).

However, when the owner was the actor, there were significant main effects of valence, *χ*^2^(1) = 14.26, *p* < .001, and judgment, *χ*^2^(1) = 412.73, *p* < .001, but the interaction was not significant, *χ*^2^(1) = 0.02, *p* = .903. As above, participants gave higher ratings for praise than blame, and these ratings were much higher for the owner (i.e., who acted) than for the dog.

As in the kite story, these findings suggest that participants were willing to give much more praise to the dog than blame. When the dog was the principal actor, the dog received much less blame than praise (*M*_difference_ = 2.83, *SE* = 0.25, Z_ratio_ = 11.32, *p* < .001). On the other hand, the owner received similar levels of vicarious blame and praise for their dog’s actions (*M*_difference_ = 0.10, *SE* = 0.25, Z_ratio_ = 0.41, *p* > .999).

#### Exploratory Analyses.

For each story, we also examined whether praise for subordinates was inflated. To do this, we focused on judgments of whether actors were responsible for their *own* actions—that is, judgments of whether the dog deserved blame or praise when it acted, and judgments of whether the owner deserved blame or praise when he acted. For both stories, 2 (valence: praise, blame) by 2 (actor: dog, owner) GEE models revealed the same pattern: a main effect of valence (kite, *χ*^2^(1) = 100.83, *p* < .001; backyard, *χ*^2^(1) = 100.53, *p* < .001), a main effect of actor (kite, *χ*^2^(1) = 30.24, *p* < .001; backyard, *χ*^2^(1) = 55.41, *p* < .001), and a significant interaction (kite, *χ*^2^(1) = 31.02, *p* < .001; *χ*^2^(1) = 51.80, *p* < .001). The interactions resulted because participants assigned owners and dog similar levels of praise for their own good actions (kite, *M*_difference_ = 0.14, *SE* = 0.19, Z_ratio_ = 0.74, *p* = .917; backyard, *M*_difference_ = 0.18, *SE* = 0.23, Z_ratio_ = 0.78, *p* = .868), but assigned much more blame to owners than to dogs for their own harmful actions (kite: *M*_difference_ = 2.03, *SE* = 0.31, Z_ratio_ = 6.48, *p* < .001; backyard, *M*_difference_ = 2.54, *SE* = 0.26, Z_ratio_ = 9.60, *p* < .001).

### Discussion

These findings again point to a double standard of ownership—owning property poses risks for blame not matched by opportunities for credit. When the dog caused harm, participants saw it and its owner as similarly blameworthy. However, when the dog was helpful, they assigned the owner much less praise than the dog. Results were completely different when the owner was the principal actor, as the owner was always assigned much more blame and praise than the dog.

The findings also cast doubt on the concern that praise for subordinates was inflated by surprise or related factors that might not apply when assigning praise to owners. After all, participants assigned similar praise to humans and dogs for their own good actions. Things were different with blame though; participants assigned much more blame to humans for their own negative actions.

## GENERAL DISCUSSION

Participants assigned owners at least as much blame as they assigned property for harm caused by the property, but they did not offer owners equal praise for their property’s good outcomes. For instance, when a pet dog caused harm, people either assigned more blame to its owner or as much blame as they assigned to the dog itself. But when the dog produced a good outcome, participants judged it deserved much more praise than its owner. These findings show a double standard of ownership—owning property poses risks for moral blame that are not balanced with equal opportunities for credit. Prior work already explored how owners are blamed when property causes harm (e.g., Bowman-Smith et al., 2018; Chiu & Hong, 1992). We extended this by examining whether people praise owners for good outcomes produced by their property, and by comparing how people assign responsibility to both owners and the property itself. The results point to a novel asymmetry between blame and praise.

Participants also assessed responsibility in two other authority-based relations, parenting and employment. With parents and children, participants showed similar patterns of praise and blame as with ownership, which suggests the double standard is not specific to ownership.^1^ With employers and employees, participants showed a different response pattern and assigned greater blame and praise to employees. So the double standard does not extend to all authority-based relations; it may only arise when subordinates are seen as lacking full autonomy and moral agency.

Why do people show the double standard of ownership? One explanation stems from the ideas that people find it more important to explain and make meaning of bad outcomes than good ones (Baumeister et al., 2001) and often look to higher authorities when explaining bad outcomes (e.g., Gray & Wegner, 2010; Haidt & Baron, 1996). Together, these tendencies might make people more likely to assign blame than praise to powerful agents. By contrast, agents like dogs, robots, and young children may be seen as poor targets for moral blame because they lack autonomy and sophisticated mental abilities (e.g., Gray et al., 2007). But people might still be willing to praise them for helpful actions because mental abilities matter less when people assign praise than blame (e.g., Guglielmo & Malle, 2019; Monroe et al., 2018; Ohtsubo, 2007).

Another explanation is that the double standard of ownership follows from an asymmetry relating to negligence. People see owners as obligated to prevent their property from causing harm, and likely see owners as negligent when (foreseeable) harm results (Malle et al., 2014). But owners are not obligated to have their property produce good outcomes (i.e., there is no equivalent of negligence for positive actions; Guglielmo & Malle, 2019), so nothing compels people to praise owners when their property does good. A similar account could be fleshed out in terms of counterfactual reasoning and causal attributions: When seeking to identify the cause of a moral violation, people may compare the events that did happen with those in counterfactual situations with no violation (Hitchcock & Knobe, 2009).^2^ In our studies, then, participants might have compared the situation where the agentic property caused harm with counterfactuals where this was avoided—perhaps counterfactuals where the owner acted to prevent it. Such comparisons would increase attributions of causality and moral responsibility to the owner, and they might likewise reduce these attributions for the agentic property (see Kominsky et al., 2015). Although participants might also have used counterfactual comparisons to attribute causality when the agentic property was helpful, the owner probably wouldn’t figure prominently in these comparisons. After all, owners aren’t expected to prevent their property from doing good. However, some of our findings might not fit with these explanations. For instance, these accounts might predict that owners will receive greater blame for harmful outcomes than praise for helpful ones. But we did not find this in the second and third experiments.

The double standard could also relate to links between causality and attributions of intentionality. When subordinates produce harm, people assign greater causal and moral responsibility to authorities if they intended for the subordinates to produce it compared with if the authorities did not intend this (Phillips & Shaw, 2015). Participants in our experiments might have inferred that the authorities had intended the harms and attributed blame accordingly. Corresponding inferences of intentionality for good outcomes might be less likely though (Knobe, 2003). This account might plausibly explain results in some of our scenarios, as when a dog injured a neighbor who unexpectedly entered the owner’s backyard. But it might be less likely for others, as when the dog wandered away from the owner and then ran away with a little boy’s teddy bear. Regardless, this account could be probed by including measures of perceived intentionality.

### Future Directions

In our experiments, participants were always asked about *both* authorities and subordinates. They were either asked which deserved more blame or praise in a single question (Experiment 1) or asked to give blame or praise ratings for both agents. (Experiments 2 and 3). These approaches might have prompted participants to compare authorities and subordinates, and so findings might differ if participants evaluated just one (see Hsee et al., 2004 for a review of ways joint versus separate evaluations can produce differing results). For example, with separate evaluations, participants may attribute greater blame to both subordinates and authorities. Seeing harm prompts people to search for an agent to blame (Cushman, 2008; Gray et al., 2012; Malle et al., 2014), and with just one agent offered as a potential target, participants might seize the opportunity. These independent judgments could be explored in future studies.

Another direction for future research will be to test whether the double standard extends to other kinds of agentic property. In our experiments, participants never assigned more blame to property than owners, a finding broadly consistent with other work showing that people blame artificial agents less than they blame humans (e.g., Maninger & Shank, 2022). But people do often assign self-driving cars more blame for traffic accidents than they assign to humans in similar accidents (e.g., Franklin et al., 2021; Hong et al., 2020; Liu et al., 2019; for a review see Bonnefon et al., 2024).^3^ So perhaps the double standard of ownership would not arise for them. Robots, self-driving cars, and other artificial agents also raise other questions. Some questions arise because the owner of an artificial agent is not the only person who can be held responsible for its conduct—with accidents involving a self-driving car, blame can also be assigned to its designer, manufacturer, or even the government (e.g., Awad et al., 2020; Copp et al., 2023; Li et al., 2016; Liu & Du, 2022). Other questions arise because robots widely vary in how human they seem and in their sophistication. This means there can be no single way people assign responsibility to them (Bigman et al., 2019). With especially sophisticated or agentic robots, people might no longer show the double standard of ownership. Instead, they might see these robots much like employees who produce bad and good outcomes. At that point, though, people might no longer see the robots as property at all.

## ACKNOWLEDGMENTS

We thank Simon Stephan and Jonathan Kominsky for helpful feedback on this work.

## FUNDING INFORMATION

This research was funded by a grant from Social Sciences and Humanities Research Council of Canada Insight Grant awarded to OF.

## DATA AVAILABILITY STATEMENT

Preregistrations, materials, data, and code for all experiments are available on OSF at https://osf.io/64zpu/. We disclose all measures, manipulations, and exclusions.

## Notes

^1^ Alternatively, participants could have seen parenting as a form of ownership. Young children appear to think they are owned, and even some adults claim this (Starmans & Friedman, 2023). So perhaps people tacitly think of young children as the property of their parents.^2^ Although we focus on violations of moral norms, counterfactual comparisons are similarly affected by violations of statistical norms (e.g., Icard et al., 2017).^3^ People do sometimes blame humans more than self-driving cars (e.g., Li et al., 2016). For example, this can happen when the driver of the vehicle actively oversees it, or operates the vehicle jointly (Awad et al., 2020; Copp et al., 2023).
